# Composition of and changes in faecal microbiota in children diagnosed with oligoarticular juvenile idiopathic arthritis

**DOI:** 10.3389/fmed.2026.1877706

**Published:** 2026-07-08

**Authors:** Esra Donmez, Dicle Sener Okur, Ata Umut Ozsoy, Hande Senol, Selcuk Yuksel

**Affiliations:** 1 Department of Pediatrics, Pamukkale University Faculty of Medicine, Denizli, Türkiye; 2 Division of Pediatric Infectious Diseases, Department of Pediatrics, Pamukkale University Faculty of Medicine, Denizli, Türkiye; 3 Diagen Biotechnological Systems and Health Inc., Ankara, Türkiye; 4 Department of Biology, Faculty of Science, Hacettepe University, Ankara, Türkiye; 5 Department of Biostatistics, Pamukkale University Faculty of Medicine, Denizli, Türkiye; 6 Division of Pediatric Rheumatology, Department of Pediatrics, Pamukkale University Faculty of Medicine, Denizli, Türkiye; 7 Division of Pediatric Rheumatology, Department of Pediatrics, Canakkale Onsekiz Mart University Faculty of Medicine, Canakkale, Türkiye

**Keywords:** children, juvenil idiopathic arthritis, microbiome, microbiota changes, rheumatology

## Abstract

**Background:**

In genetically predisposed individuals, changes in the balance of pro- and anti-inflammatory bacteria in the intestinal microbiota may affect the mucosal immune system and contribute to the development of Juvenile Idiopathic Arthritis (JIA). In this study, we aimed to compare the bacterial composition of the faecal microbiota between children with newly diagnosed, untreated oligoarticular JIA and healthy children, in Türkiye.

**Materials and methods:**

This study included 25 healthy children and 25 treatment-naive patients diagnosed with oligoarticular JIA. Targeted sequencing of the bacterial 16S ribosomal RNA (rRNA) gene was performed on the genetic material obtained from stool samples.

**Results:**

Diversity analyses revealed that the dominant bacteria were present in similar proportions, and their distributions were similar. The number of rare species differed, and their distributions were heterogeneous. The patient group was found to have a higher abundance of Bacteroidetes and a lower Firmicutes/Bacteroidetes ratio. The relative abundance of Dialister was reduced, while Oscillibacter and Alistipes were increased in the patient group. In the patient group, we noted an increase in the Akkermansiaceae family and in the Catenibacterium, Howardella, Holdemanella, Megasphaera and Akkermansia genera, a decrease in the Clostridiaceae and Lactobacillaceae families and Lactobacillus genera.

**Conclusion:**

To the best of our knowledge, our study is the first of its kind conducted on this subject in Türkiye. Given that microbiota composition is influenced by geographical characteristics, our study also contributes to the literature regarding the faecal composition of JIA patients in our country. Funding Scientific Research Projects Unit of Pamukkale University.

## 1 Introduction

The gut microbiota composition in individuals with various chronic conditions such as inflammatory bowel diseases (IBD), rheumatoid arthritis (RA), enthesitis-related arthritis (ERA), ankylosing spondylitis (AS), psoriatic arthritis (PsA), systemic lupus erythematosus (SLE), Graves’ disease and type 1 diabetes differs from that of healthy individuals (1–9).

The intestinal mucosa limits the access of gut bacteria to lymphoid tissues and prevents the dysregulation of the local innate and adaptive immune systems (10). Short-chain fatty acids (SCFAs)—such as butyrate, propionate and acetate—produced by certain bacteria in the gut microbiota exert significant immunomodulatory effects through various mechanisms, including inducing the differentiation of regulatory T cells, increasing interleukin-10 (IL-10) production and suppressing T helper 17 (Th17) cells, thereby reducing pro-inflammatory factor production (11–18).

Juvenile idiopathic arthritis (JIA) is the most common rheumatic disease in childhood, and it has been suggested that environmental and genetic factors play a role in its aetiology and pathogenesis (16–20).

In genetically predisposed individuals, it is thought that changes in the balance of pro- and anti-inflammatory bacteria in the intestinal microbiota affect the mucosal immune system and contribute to the development of JIA (21–23).

It has been suggested that faecal microbial diversity is reduced and its composition differs in children with JIA and that these may lead to subclinical intestinal inflammation, thereby triggering joint inflammation (24, 25). In JIA patients with gastrointestinal (GI) symptoms but without IBD, mucosal changes indicative of intestinal inflammation—such as increased numbers of small intestinal intraepithelial T cells and cytotoxic lymphocytes and increased human leukocyte antigen – DR isotype (HLA-DR) expression in the ileal mucosa—have been observed, regardless of disease category (26). Evidence has been presented that the faecal microbiota of children with JIA contains higher levels of mucin-degrading *Bacteroides* and *Akkermansia muciniphila (A. muciniphila)*, which are important components of the primary mucosal defence (27). The breakdown of mucin increases bacterial access to the intestinal immune system, triggering an inflammatory process (10, 28–31). These findings support the hypothesis of an association between the gut microbiota and JIA (12, 32).

In this study, we aimed to compare the bacterial composition of the faecal microbiota between children with newly diagnosed, untreated oligoarticular JIA and healthy children to determine which bacteria play a role in the development of JIA in children, especially in our country of Türkiye.

## 2 Materials and methods

**Figure 1:**
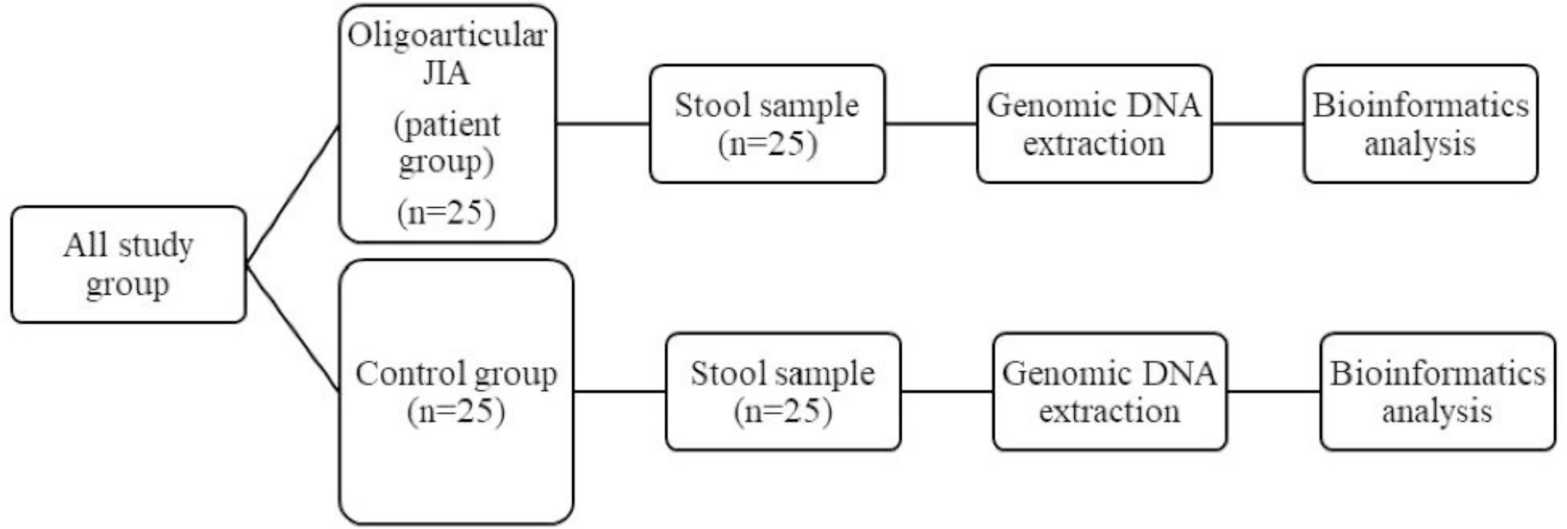
Flow diagram of the study.

This prospective study included, 25 control subjects and 25 treatment-naive patients diagnosed with oligoarticular JIA, at the Department of Paediatric Rheumatology, Pamukkale University Faculty of Medicine, with ages ranging from 25 months to 188.5 months, between 2022 and 2024. Patients who were regularly taking medication, had used antibiotics and/or probiotics within the past 12 weeks or had other autoinflammatory, autoimmune or chronic conditions were excluded from the study (Figure 1).

Approval was obtained from the Pamukkale University Faculty of Medicine Clinical Research Ethics Committee via decision no. E-60116787-020-299011 dated November 29, 2022. The study was supported by the Scientific Research Projects Unit of Pamukkale University, project no. 2023TIPF008.

### 2.1 Data collection

Following the acquisition of parental consent for children in both the patient and control groups who met the inclusion criteria, stool samples were collected in transport media. These were then transferred to −80 °C for storage in an upright position until deoxyribonucleic acid (DNA) extraction. The age, sex, mode of delivery, birth weight, feeding history and methods, height and weight at the time of presentation of all children and symptoms of the children in the patient group were recorded. Pathological findings identified during the physical examination were also recorded. Patients’ inflammation biomarkers [erythrocyte sedimentation rate (ESR), ferritin, C-Reactive Protein (CRP)], anti-nuclear antibody (ANA), rheumatoid factor (RF) and HLA-B27 results and Juvenile Arthritis Disease Activity Scores (JADAS) were examined on the date the stool sample was collected (Figure 1).

### 2.2 Analysis of stool samples

#### 2.2.1 DNA extraction from stool samples

The QuickGene extraction system and DNA tissue extraction kit (Kurabo Industries Ltd., Osaka, Japan) were used for the extraction protocol. Briefly, 25 mg of stool sample was transferred into a homogenisation tube along with 250 μL of MDT (Tissue Lysis) solution supplied with the QuickGene kit. To ensure thorough homogenisation, either 15 mg of 0.1-mm diameter glass beads or 10 pieces of 1.0-mm diameter zircon beads were added to the tube. Homogenisation was conducted at 5,000 rpm for 2 × 120 s. Following homogenisation, 25 μL of EDT (Proteinase K) solution was added, and the sample was incubated at 56 °C for 60 min. The sample was then centrifuged at 15,000 × *g* for 10 min at room temperature. After centrifugation, 200 μL of the supernatant was transferred to a 1.5-mL microtube. Subsequently, 180 μL of LDT (Cell Lysis) solution was added, and the contents were mixed for 15 s before incubating the microtube at 70 °C for 10 min. In the next step, 240 μL of ice-cold 99% ethanol was added followed by 15 s of mixing. The entire contents of the microtube were then loaded onto a QuickGene filter cartridge (Kurabo Industries Ltd., Osaka, Japan), and the washes were performed according to the instrument protocol. Three wash steps were conducted using 750 μL of WDT (Wash Buffer) solution per wash. At the end of the extraction procedure, an average of 50–60 ng of genomic DNA was obtained, eluted in 200 μL of CDT (Elution Buffer) (Figure 1).

#### 2.2.2 16S ribosomal RNA sequencing analysis

Targeted sequencing of the bacterial 16S ribosomal RNA (rRNA) gene was performed on the genetic material obtained in this study. The V3–V4 hypervariable region of the bacterial 16S rRNA gene was amplified using Illumina overhang adapter-linked locus-specific primers targeting the 341F/805R region, according to the 16S Metagenomic Sequencing Library Preparation protocol (Illumina, Part #15044223 Rev. B). The amplicon libraries were cleaned by size selection of larger fragments using AMPure XP beads (Beckman Coulter, Brea, CA, USA), after which they were normalised and pooled. Once the library pool was prepared, sequencing was performed on the MiSeq platform (Illumina Inc., San Diego, CA, USA).

#### 2.2.3 Bioinformatics analysis

Raw sequencing data were analysed using the QIIME 2 platform (https://qiime2.org; accessed on 16 February 2025). In the first step, demultiplexing was performed to separate the reads assigned to each sample. Quality filtering, error correction and chimera detection were then conducted using the DADA2 algorithm implemented in QIIME 2 (https://benjjneb.github.io/dada2/; accessed on 16 February 2025). During this process, low-quality bases (Q-score <25) and short reads (<200 bp) were removed. Amplicon sequence variants (ASVs) were identified through the DADA2 pipeline. Taxonomic classification of each ASV was performed using a naive Bayes classifier trained on the SILVA 138.1 16S rRNA gene reference database (https://www.arb-silva.de; accessed on 16 February 2025), and classification results were obtained at the phylum, class, order, family and genus levels. Putative species-level assignments are reported only as tentative taxonomic assignments because 16S V3–V4 sequencing has limited resolution for definitive species-level identification. For alpha diversity analyses, the Shannon index, Faith’s phylogenetic diversity, Chao-1 index, Fisher’s alpha, observed ASV count, Pielou’s evenness index and Simpson’s index were calculated. Between-group comparisons of alpha diversity were conducted using the Kruskal–Wallis test. Beta diversity analyses employed the Bray–Curtis, Jaccard, unweighted UniFrac and weighted UniFrac distance metrics. Principal coordinates analysis (PCoA) ordinations were visualised using EMPeror (https://github.com/biocore/emperor; accessed on 16 February 2025). Between-group differences in beta diversity were assessed with permutational multivariate analysis of variance (PERMANOVA), permutational analysis of multivariate dispersions (PERMDISP) and analysis of similarities (ANOSIM) tests. Differential taxonomic abundance between groups was analysed using ANCOM-BC (Analysis of Compositions of Microbiomes with Bias Correction; https://bioconductor.org/packages/ANCOMBC; accessed on 16 February 2025).

This method accounts for the inherent characteristics of microbiome compositional data, including sparsity and compositionality, while controlling the false-positive rate.

Analyses were performed at the phylum, class, order, family and genus levels, and results were visualised using volcano plots. Results of taxonomic composition analyses were presented through bar charts and heatmaps. Detailed taxonomic hierarchies and abundance distributions for each sample were displayed interactively using Krona charts (https://github.com/marbl/Krona; accessed on 16 February 2025). All visualisations and statistical analyses were performed using R (https://www.r-project.org; accessed on 16 February 2025) and Python (https://www.python.org; accessed on 16 February 2025) (Figure 1).

### 2.3 Statistical methods

Statistical analyses for the evaluation of demographic and laboratory characteristics of the patient and control groups were performed using the Statistical Package for the Social Sciences version 25.0 [IBM SPSS Statistics 25 software (SPSS; IBM Corp., Armonk, NY, USA)]. In addition to descriptive statistics, Pearson’s chi-square test, the Kruskal–Wallis test and a one-way analysis of variance (ANOVA) were used to compare qualitative data and quantitative data, as appropriate. A *p*-value of less than 0.05 was considered statistically significant. Microbiota analyses were evaluated using R software. Potential confounders, including age, sex, diet, body mass index/nutritional status, mode of delivery and breastfeeding history, were evaluated descriptively and compared between groups where available. Because of the limited sample size and the number of covariates relative to the number of participants, multivariable confounder-adjusted microbiota models were not performed. Therefore, the microbiota findings should be interpreted as unadjusted associations. The substantial age imbalance between groups was specifically considered in the interpretation of the results and is acknowledged as a major limitation.

## 3 Results

### 3.1 Demographic and clinical data

The study included a total of 50 children [22 boys (44%), 28 girls (56%)], comprising 25 children with oligoarticular JIA (9 boys, 16 girls) and 25 control subjects (13 boys, 12 girls). The median age in the patient group and the control group was 150 months (108–188.5) and 50 months (25–104), respectively. Although the age distributions of the patient and control groups were found to be statistically significantly different, both groups were distributed above the age of 2 years (*p* = 0.0001). No statistically significant differences were found between the study groups in terms of mode of delivery, gestational age at birth, birth weight, duration of breastfeeding (*p* = 0.254, *p* = 0.609, *p* = 0.101, *p* = 0.807). Most of the children in both the patient and control groups lived in the Aegean region; the geographical distribution of children in both groups was similar (*p* = 0.239). When vegetable, carbohydrate and meat consumption was assessed, the dietary patterns of the patient and control groups were similar (*p* = 0.671) (Table 1). Examination of the patients’ laboratory results revealed that haemogram and acute phase reactants were within normal limits (Table 2).

**Table 1:** Demographic and clinical data.

Characteristics	Patient (*n* = 25)			Control (*n* = 25)			*p*
Mode of delivery	C/S	NVYD		C/S	NSVD		*p* = 0.254
	12 (48%)	13 (52%)		16 (64%)	9 (36%)		
Gestational age at birth	Preterm	Term		Preterm	Term		*p* = 0.609
	1 (4%)	24 (96%)		3 (12%)	22 (88%)		
Birth weight (g)	0–2,500	2,500–4,000	>4,000	0–2,500	2,500–4,000	>4,000	*p* = 0.101
	0 (0%)	23 (%92)	2 (8%)	3 (12%)	21 (84%)	1 (4%)	
Duration of breastfeeding	<6	6–12	>12	<6	6–12	>12	*p* = 0.807
	2 (8%)	6 (24%)	17 (68%)	8 (32%)	6 (24%)	11 (44%)	

C/S, cesarean section; NSVD, normal spontaneous vaginal delivery; g, gram.

**Table 2:** Laboratory results, JADAS scores of the patient group.

Laboratory parameters	Median (IQR)
WBC (/mm^3^)	7,300 (6,005–8,450)
PLT (/mm^3^)	338,000 (278,500–383,500)
ESR (mm/h)	22 (8–45)
CRP (mg/l)	1,1 (0,57–13)
Fibrinojen (mg/dl)	305 (247,5–400,5)
JADAS27	14 (10–25,5)

IQR, interquartile range; WBC, white blood cell; PLT, platelet; ESR, erythrocyte sedimentation rate; CRP, C reactive protein; JADAS, Juvenile Arthritis Disease Activity Score.

### 3.2 Evaluation of intestinal microbiota

#### 3.2.1 Alpha diversity

**Figure 2:**
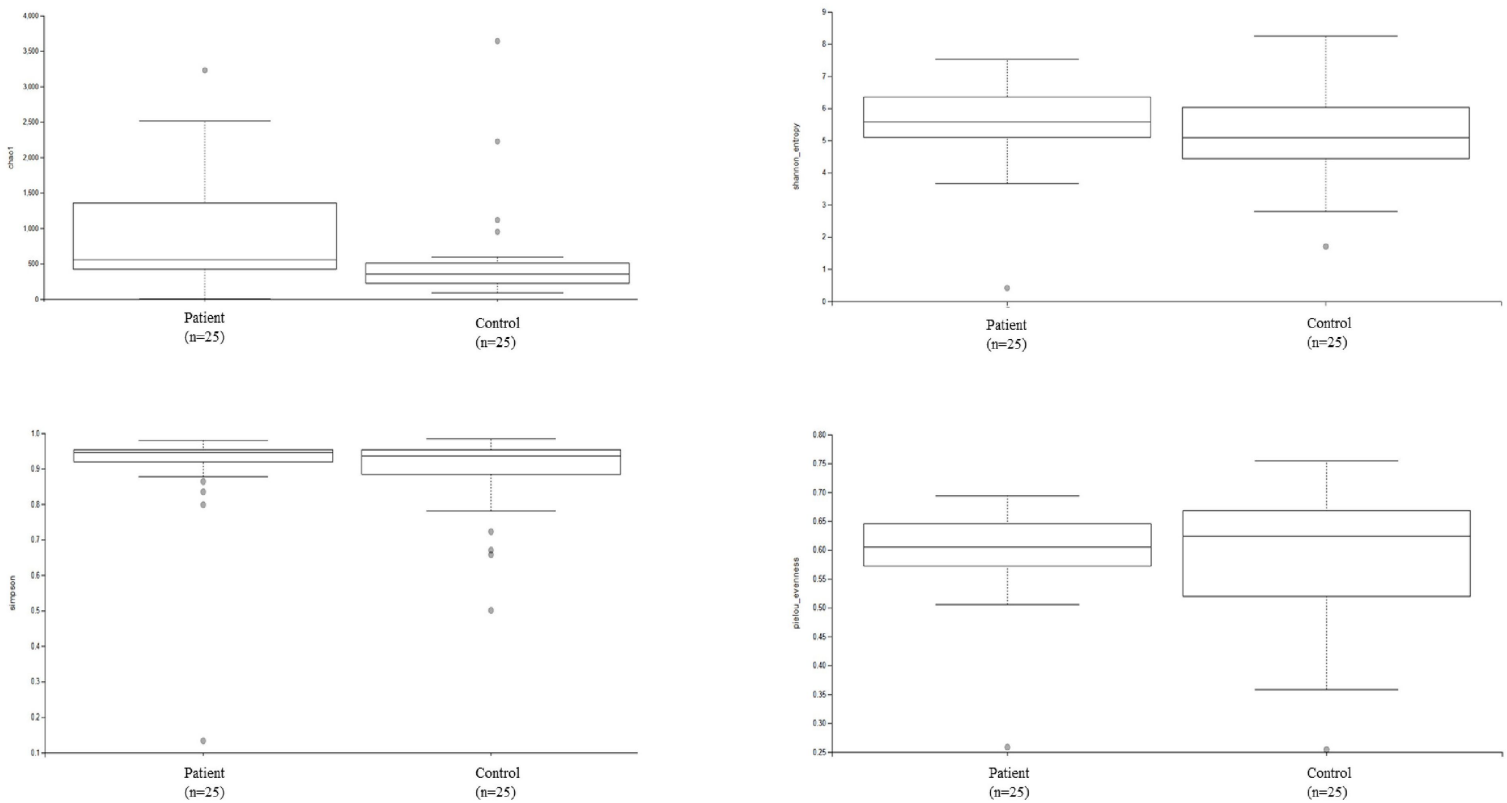
Comparison of alpha diversity index of groups.

While a statistically significant difference was observed in the comparison of alpha diversity using the Chao-1 index between the patient and control groups (*p* < 0.004), no significant difference was found in the comparisons using the Shannon index or Simpson index (*p* = 0.06, *p* = 0.33) In the Pielou’s evenness comparison, no statistically significant difference was found between the patient and control groups (*p* = 0.62) (Figure 2).

#### 3.2.2 Beta diversity

**Figure 3:**
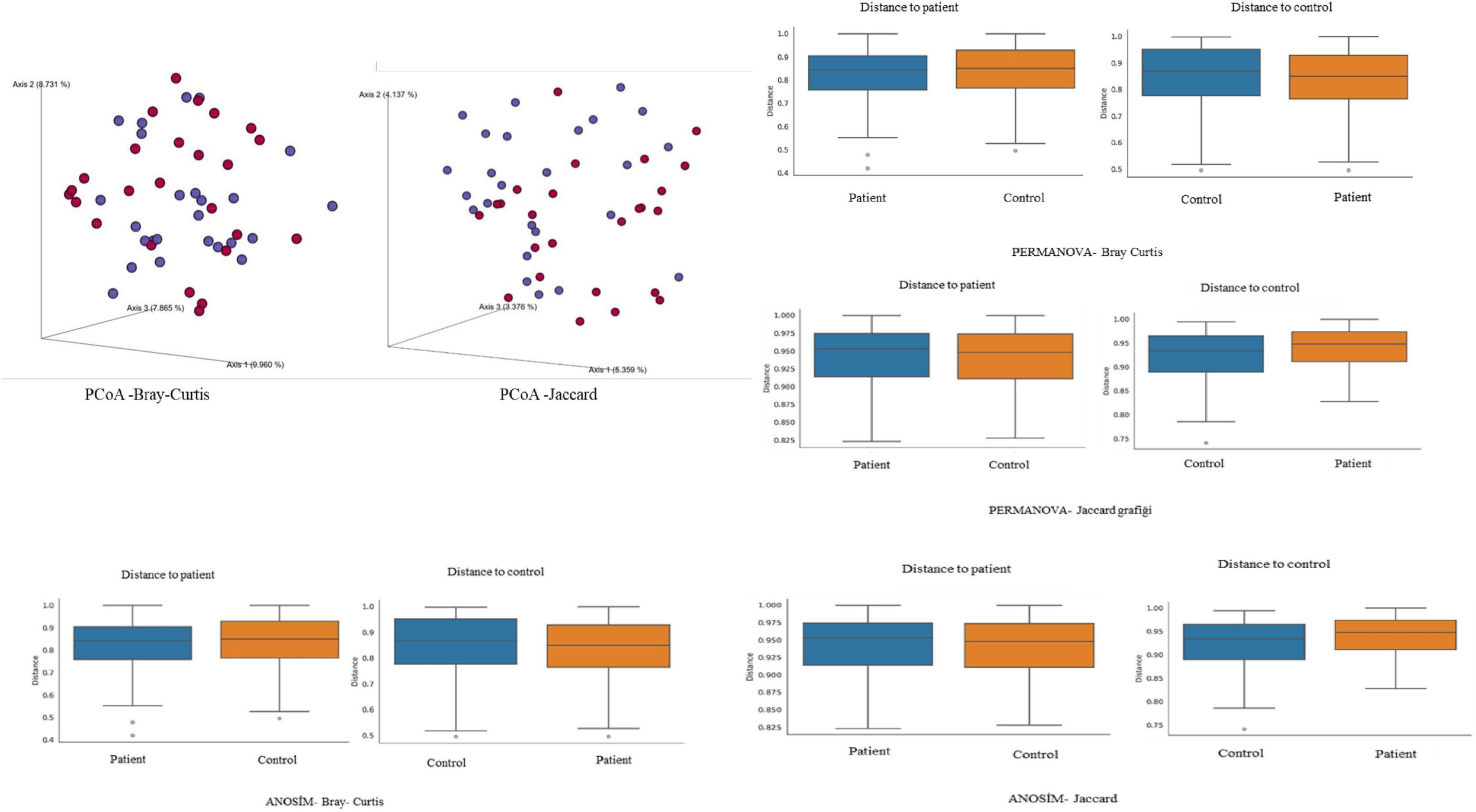
Comparison of beta diversity index of groups.

When the Bray-Curtis distance metric was used in the PERMANOVA test for beta diversity analysis, no statistically significant difference was detected between the patient and control groups (*p* = 0.425). In the PERMANOVA test, when the Jaccard distance was used, a statistically significant difference in microbial community structure was detected between the two groups (*p* = 0.002). In the PERMDISP test, which compares differences in microbial community distribution, no statistically significant difference was detected in species distribution between the patient and control groups when the Bray-Curtis and Jaccard distances were used (*p* = 0.558, *p* = 0.231). When the groups were compared in the PCoA, it was found that both groups clustered in similar areas. It was therefore demonstrated that there was no clear distinction between the control group and the patient group in terms of microbiota community structures. When the Bray-Curtis distance was used in the ANOSIM test to compare the microbial composition between the patient and control groups, no statistically significant difference was detected (*R* = −0.0088, *p* = 0.639). However, when the Jaccard distance was used in the ANOSIM test, a statistically significant difference was detected (*R* = 0.074, *p* = 0.002) (Figure 3). The ANOSIM test results indicated that there were qualitative differences in microbial composition between the groups but that these differences were not reflected in abundance levels.

#### 3.2.3 Comparison of ASVs

**Figure 4:**
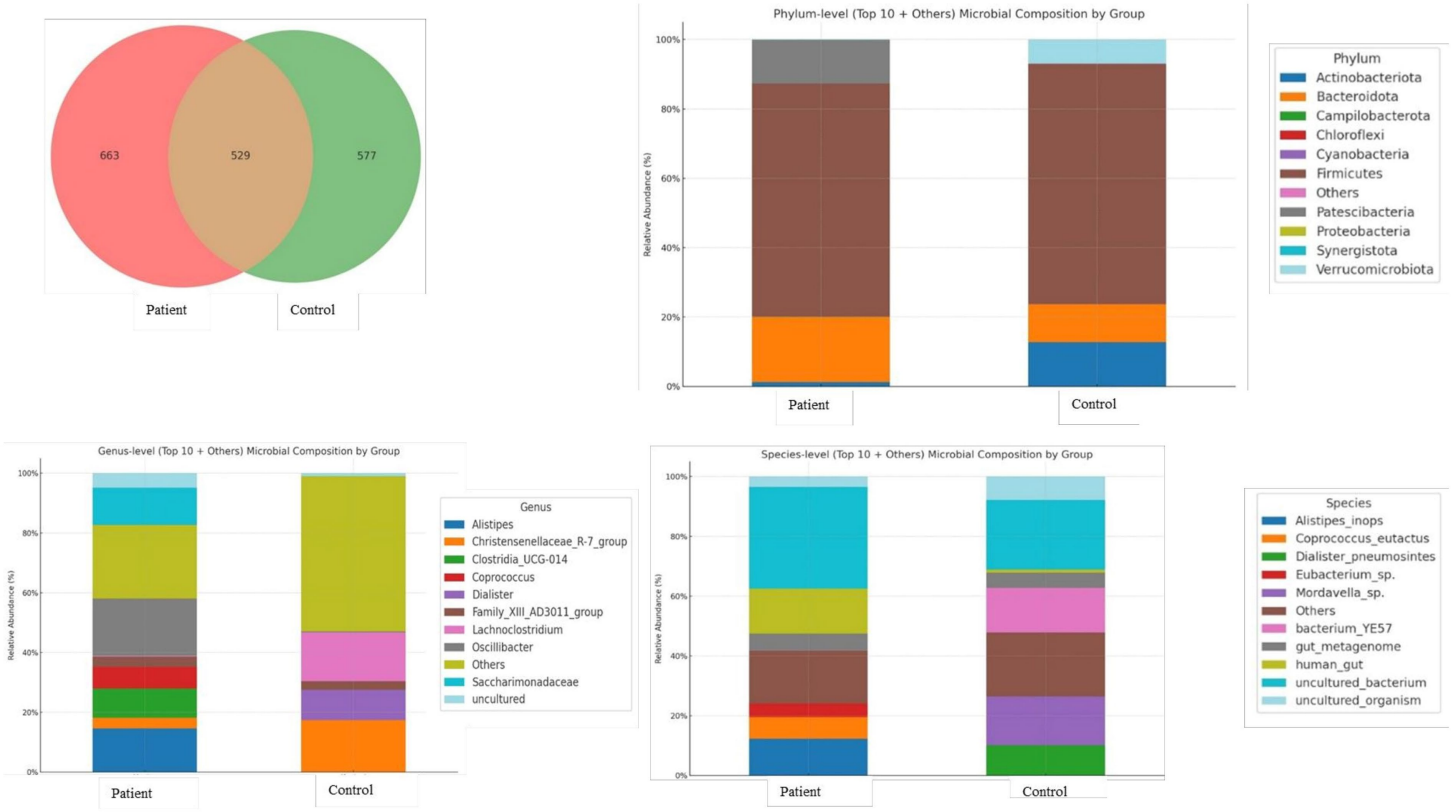
OTUs and comparison of faecal microbiota composition.

To better understand the distribution of bacteria within the microbiota, the ASVs shared between the patient and control samples were analysed. Of the total 1,769 ASVs, 577 were found only in the control group, 663 were found only in the patient group and 529 were shared between the two groups (Figure 4).

#### 3.2.4 Microbiota composition

##### 3.2.4.1 Descriptive relative-abundance results

Firmicutes, Bacteroidota, Patescibacteria, Actinobacteria and Verrucomicrobia were the most dominant phyla. The Firmicutes/Bacteroidota ratio was found to be 3.57 in the patient group and 6.34 in the control group. In the patient group, Firmicutes (67.2%), Bacteroidota (18.82%), Patescibacteria (12.53%), Actinobacteria (1.21%), Verrucomicrobia (0.06%), Proteobacteria (0.04%), Chloroflexi (0.05%), Campylobacterota (0.03%) and Synergistota (0.02%) were detected. In the control group, Firmicutes (69.25%), Bacteroidota (10.92%), Actinobacteria (12.73%), Verrucomicrobia (6.95%), Cyanobacteria (0.12%) and Patescibacteria (0.01%) were detected (Figure 4).

In terms of the composition of the faecal microbiota at the genus level, *Oscillibacter* (19.2%), *Alistipes* (14.59%), *Saccharimonadaceae* (12.53%), *Clostridia UCG 014* (9.74%), *Coprococcus* (7.27%), *Christensenellaceae* (3.58%), *Lachnoclostridium* (0.24%) and *Dialister* (0.03%) were identified in the patient group. In the control group, *Christensenellaceae* (17.35%), *Lachnoclostridium* (16.3%), *Dialister* (10.12%), *Alistipes* (0.01%), *Clostridia UCG 014* (0.03%) and *Oscillibacter* (0.25%) were detected (Figure 4).

Putative species-level taxonomic assignments suggested that *Alistipes inops (A.inops)* (12.27%), *Coprococcus eutactus (C.eutactus)* (7.27%), *Eubacterium* sp. (4.47%) and *bacterium YE57* (0.1%) were detected in the patient group, while *Dialister pneumosintes (D.pneumosintes)* and *Mordavella* sp. were not. In the control group, *Mordavella* sp. (16.28%), *bacterium YE57* (14.86%), *D.pneumosintes* (10.12%), and *Eubacterium* sp. (0.02%) were detected (Figure 4).

##### 3.2.4.2 ANCOM-BC based evaluation of bacterial taxa

**Figure 5:**
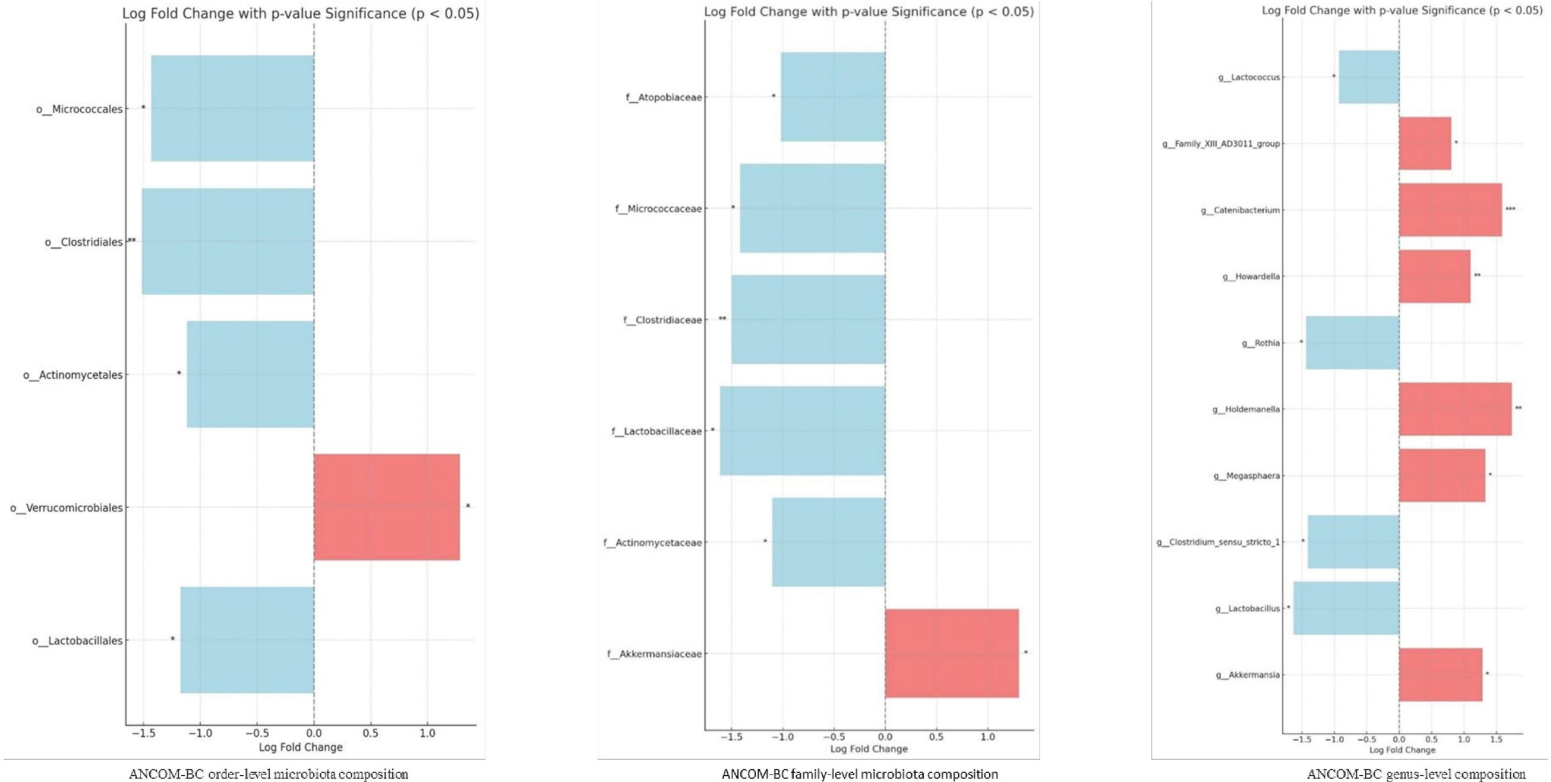
ANCOM-BC order, family, genus level microbiota composition.

Using the ANCOM-BC method, statistically significant differences were observed between the patient and control groups at the order, family and genus levels (Figure 5).

In the patient group, compared with the control group, an increase was observed in the Verrucomicrobiales order (LFC 1.28), while decreases were noted in the Micrococcales (LFC −1.43), Clostridiales (LFC −1.51), Actinomycetales (LFC −1.11) and Lactobacillales (LFC −1.17) orders (Table 3).

**Table 3:** ANCOM-BC identified differential abundance analysis results in the study group.

Taxonomic level	Taxon	Patient group	Control group	LFC
Order	Verrucomicrobiales	↑ Increased	↓ Decreased	1.28
	Micrococcales	↓ Decreased	↑ Increased	−1.43
	Clostridiales	↓ Decreased	↑ Increased	−1.51
	Actinomycetales	↓ Decreased	↑ Increased	−1.11
	Lactobacillales	↓ Decreased	↑ Increased	−1.17
Family	Akkermansiaceae	↑ Increased	↓ Decreased	1.301
	Atopobiaceae	↓ Decreased	↑ Increased	−1.01
	Micrococcaceae	↓ Decreased	↑ Increased	−1.41
	Clostridiaceae	↓ Decreased	↑ Increased	−1.49
	Lactobacillaceae	↓ Decreased	↑ Increased	−1.61
	Actinomycetaceae	↓ Decreased	↑ Increased	−1.102
Genus	*Catenibacterium*	↑ Increased	↓ Decreased	1.58
	*Family XIII AD3011 group*	↑ Increased	↓ Decreased	0.79
	*Howardella*	↑ Increased	↓ Decreased	1.10
	*Holdemanella*	↑ Increased	↓ Decreased	1.73
	*Megasphaera*	↑ Increased	↓ Decreased	1.32
	*Akkermansia*	↑ Increased	↓ Decreased	1.28
	*Lactococcus*	↓ Decreased	↑ Increased	−0.93
	*Rothia*	↓ Decreased	↑ Increased	−1.43
	*Clostridium sensu stricto 3*	↓ Decreased	↑ Increased	−1.40
	*Lactobacillus*	↓ Decreased	↑ Increased	−1.63

LFC, Log fold change. Positive LFC values indicate increased abundance in the patient group, while negative LFC values indicate decreased abundance.

In the patient group, compared with the control group, an increase was observed in the Akkermansiaceae (LFC 1.301) family, while decreases were observed in the, Atopobiaceae (LFC −1.01), Micrococcaceae (LFC −1.41), Clostridiaceae (LFC −1.49), Lactobacillaceae (LFC −1.61) and Actinomycetaceae (LFC −1.102) families (Table 3).

In the patient group, compared to the control group, increases were observed in the genera *Catenibacterium* (LFC 1.58), the *Family_ XIII_AD3011 group* (LFC 0.79), *Howardella* (LFC 1.10), *Holdemanella* (LFC 1.73), *Megasphaera* (LFC 1.32) and *Akkermansia* (LFC 1.28), while decreases were observed in the genera *Lactococcus* (LFC −0.93), *Rothia* (LFC −1.43), *Clostridium sensu stricto 3* (LFC −1.40) and *Lactobacillus* (LFC −1.63) (Table 3).

## 4 Discussion

A total of 50 children were included in our study, comprising 25 treatment-naive children with oligoarticular JIA and 25 control subjects. No significant differences were observed between the patient group and the control group in terms of demographic data, apart from age.

The median age in the patient group and the control group was 150 months (108–188.5 months) and 50 months (25–104 months), respectively. Although the age distributions of the patient and control groups were found to be statistically significantly different, the median age in both groups was over 2 years, and both groups showed a distribution above 2 years of age. Given that the faecal microbiota develops to a degree resembling the adult microbiota from birth to 2 years of age and stabilises in a manner similar to that of adults after this age. This difference was taken into account when interpreting the analysis results and needs to be clarified through studies with larger, age-matched cohorts (28, 33–35).

In our study, a statistically significant difference was observed between the patient and control groups in the comparison of the Chao-1 index, and it was found to be higher in the former. The Chao-1 index did not show a significant difference between groups in stool samples taken from 75 children diagnosed with JIA and 25 controls (28). A comparison of the Chao-1 index in stool samples from 39 JIA patients and 42 healthy children revealed a statistically significant difference, with it reported to be lower in the former compared to the latter (16). In a study involving 17 active and 15 inactive systemic-onset JIA (SoJIA) patients and 32 healthy children, it was reported that the Chao-1 index was significantly higher in both the active-SoJIA and inactive-SoJIA groups compared to the control group (36). In a Finnish study, no statistically significant difference in the Chao-1 index was observed between the JIA group and the control group (22). In our study, no statistically significant difference was found between the patient and control groups when comparing the Shannon index (*p* = 0.06). In the literature, when the Shannon index is examined, some studies report no statistically significant difference between JIA patients and controls; however, in a study involving 42 children with JIA and 40 healthy children, it was reported that the Shannon index was significantly lower in the former compared to the latter (6, 22, 28, 36). In our study, no statistically significant difference was found in the Simpson index comparison between the patient and control groups. No statistically significant difference was found in the Simpson index in a study comparing the faecal microbiota of children with SoJIA and healthy children (36). In the literature, studies examining the gut microbiota of patients diagnosed with JIA and controls reported that there was no statistically significant difference in the Simpson index comparison (16, 22).

The fact that there was a difference in the Chao-1 index but no difference in the Shannon or Simpson indices indicated that the microbial communities in the study groups differed in terms of species richness but were similar in terms of the balance of diversity distribution and the difference in the number of bacterial species between the groups was attributable to rare species. It was found that the groups contained dominant bacteria in similar proportions; the dominant species and their distributions were similar, the core microbiota was similar, the number of rare species differed.

In our study, no statistically significant difference was observed between the groups when the Bray-Curtis index was used in the PERMANOVA analysis. In contrast, a statistically significant difference was detected between the patient and control groups when the Jaccard index was used. In other words, while no statistically significant difference was detected in species abundance when the two groups were compared, one was detected in terms of species presence/absence. In a study involving 40 patients with JIA and 42 healthy children, when the Bray-Curtis distance was used, a statistically significant difference was found between the groups (16). Use of the Bray-Curtis distance revealed statistically significant differences among children with ERA, children with non-ERA (nERA) and healthy children (24). When the faecal microbiota of children with JIA and healthy children were compared in terms of beta diversity, JIA samples tended to be slightly higher than controls, although this difference was not statistically significant (22). A beta diversity analysis showed that both SoJIA groups (active and inactive) were more similar to each other than to the control group; however, when the active SoJIA group was compared with the control group, a statistically significant difference was observed between the groups (36). When our beta diversity analyses were evaluated, it was observed that there were differences between groups in terms of the presence or absence of species but that their abundances and the proportions of dominant bacteria were similar. The abundance-based composition variation among the groups was similar, whereas the variation in their distribution in terms of species presence differed, particularly as the distribution of rare species was heterogeneous.

In our study, Firmicutes and Bacteroidetes were found to be the two most common phyla; the Bacteroidetes phylum was more prevalent in the patient group (18.82%) than in the control group (10.92%), while the Firmicutes phylum was similar in both groups (67.2% vs. 69.25%); the Firmicutes/Bacteroidetes ratio was higher in the control group (6.34) compared to the patient group (3.57). Patescibacteria (12.53% vs. 0.01%) were more abundant in the patient group, while Actinobacteria (1.21% vs. 12.73%) and Verrucomicrobia (0.06% vs. 6.9%) were more abundant in the control group. Proteobacteria were detected only in the patient group (0.04%). The bacteria in the human GI microbiota belong to four main phyla, with 80%–90% belonging to Firmicutes and Bacteroidetes and 10%–20% belonging to Proteobacteria and Actinobacteria (15). As in this study, it has been reported that Firmicutes and Bacteroidetes are the most dominant phyla in children with JIA, as well as in children and adults with spondyloarthritis (spA) and RA and in control groups (15, 16, 19, 22, 24, 36–41). Numerous studies have shown that the Bacteroidetes phylum is increased in stool samples from children with ERA, JIA, spA and SoJIA compared with controls (22, 36, 42–45). In the US, when children with ERA were divided into clusters, Bacteroidetes was found to be significantly higher in one cluster compared with controls (42). It has been reported that Bacteroidetes is present in high abundance in the gut microbiota of children with JIA both before treatment and between treatment periods, as well as in patients with refractory RA compared to controls (25, 41). *Bacteroides fragilis (B.fragilis)* was found to be four times more abundant in the gut microbiota of children with ERA compared to controls, while no differences were observed in other species. It was found that patients with SpA who also had arthritis had higher levels of Bacteroidetes in their gut microbiota compared to patients with SpA who did not have arthritis (8). When the gut microbiota of children with SoJIA were compared with those of healthy children, the highest abundance of Bacteroidetes was observed in the active SoJIA group, followed by the inactive SoJIA group and finally the control group (36). However, studies involving adults with autoimmune diseases such as RA and SpA have reported that the abundance of Bacteroidetes in the gut microbiota does not increase but decreases compared to controls (7, 43, 46–51). The differing results obtained from children and adults suggest that while Bacteroidetes may not be directly linked to inflammation in autoimmune arthritis, it may contribute to a predisposition to arthritis by influencing the development of the immune system. The literature also emphasises that in patients with JIA and SoJIA, the Firmicutes/Bacteroidetes ratio is more significant in the development of arthritis than the abundance of Bacteroidetes and/or Firmicutes bacteria (22, 36, 52, 53). It has been reported that this ratio is lower in patients with difficult-to-treat RA compared to those with easily treatable RA (41). However, geographical differences should be considered when interpreting the results (22, 36, 52, 53). In our study, the higher abundance of Bacteroidetes in the patient group and the lower Firmicutes/Bacteroidetes ratio in the patient group are consistent with the literature. A limited number of studies indicate an association between the phyla Actinobacteria, Verrucomicrobia and Proteobacteria and JIA and autoimmune diseases. Qian et al., found higher levels of Proteobacteria and lower levels of Verrucomicrobia in children with JIA compared to a control group (16). Dong et al. reported a negative correlation between Proteobacteria and disease activity in children with SoJIA (16). Zhang et al., however, found that Proteobacteria was reduced in patients with RA compared to controls (38). It has been shown that Actinobacteria is increased in Finnish children with JIA and decreased in US patients with ERA (22, 43). Further studies involving different populations are needed to elucidate the relationship between these bacteria and JIA and other autoimmune arthritides.

In our study, at the genus level, *Oscillibacter* (19.2% vs. 0.25%), *Alistipes* (14.59% vs. 0.01%) and *Clostridia UCG 014* (9.74% vs. 0.03%) were found in higher abundance in the patient group, while *Christensenellaceae* (3.58% vs. 17.53%), *Lachnoclostridium* (0.24% vs. 16.53%) and *Dialister* (0.03% vs. 10.12%) were found in higher abundance in the control group. *Saccharimonadaceae* (12.53%) and *Coprococcus* (7.27%) were observed only in the patient group.

At the highest reliable taxonomic resolution based on 16S rRNA gene sequencing (V3–V4 region), *A.inops* (12.27%) and *C.eutactus* (7.27%) were detected only in the patient group, while *Mordavella* sp. (16.28%) and *D.pneumosintes* (10.12%) were detected only in the control group; *Eubacterium* sp. (4.47% vs. 0.02%) was more abundant in the patient group. Twelve genera distinguishing JIA patients from controls were identified: *Bifidobacterium*, *Lachnospira*, *Dialister*, *Roseburia*, *Oscillibacter*, *Akkermansia*, *Clostridium*, *Faecalibacterium*, *Bilophila*, *Coprococcus*, *Haemophilus* and *Anaerostipes*. It has been reported that, compared to controls, *Oscillibacter* levels are increased and those of *Dialister* and *Lachnospira* are decreased in JIA patients (16). It has been demonstrated that *Oscillibacter* levels were increased, while those of *Dialister*, *Lachnoclostridium* and *Christensenellaceae* were decreased in a JIA group compared to controls (22). It is noted that *Dialister* is a propionate-producing bacterium and may play a protective role against JIA (16, 22, 54). Of 17,055 infants whose stool samples were examined in Sweden, 111 developed JIA; it was reported that levels of *Coprococcus* and *Dialister* were lower in the group of infants who developed JIA compared to those in the other group (55). The same study reported that *Lachnoclostridium* was detected more frequently in the control group than in the patient group, while *Saccharimonadaceae* was more prevalent in the patient group (55). It has also been reported that *Coprococcus* levels are elevated in the gut microbiota of patients with SpA (56).

In recent years, changes in *Alistipes* abundance have been investigated in humans and mouse models, and the genus has been implicated in various diseases such as liver fibrosis, colorectal cancer, cardiovascular diseases and mood disorders (57–60). Fermentation of proteins in the GI tract by the gut microbiota can disrupt gut homeostasis, with *Alistipes* being one of the genera implicated in causing dysbiosis in this manner (61). In our study, consistent with the literature, we observed a decrease in *Dialister*, *Lachnoclostridium* and *Christensenellaceae* and an increase in *Oscillibacter*, *Saccharimonadaceae* and *Alistipes* in children with JIA. As in most studies, *Coprococcus* was also increased in the patient group in our study; however, studies have also reported different results. This requires further clarification through more comprehensive studies involving different populations.

In our study, the ANCOM-BC differential abundance analysis revealed an increase in the Verrucomicrobiales order and a decrease in the Micrococcales, Clostridiales, Actinomycetales and Lactobacillales orders in children with JIA. It also showed an increase in the Akkermansiaceae family and a decrease in the Atopobiaceae, Micrococcaceae, Clostridiaceae, Lactobacillaceae and Actinomycetaceae families. Finally, it revealed an increase in the genera *Catenibacterium*, *Family_ XIII_AD3011 group*, *Howardella*, *Holdemanella*, *Megasphaera* and *Akkermansia* and a decrease in the genera *Lactococcus*, *Rothia*, *Clostridium sensu stricto 1* and *Lactobacillus*.

First identified in 2004, *A. muciniphila* sustains its life by consuming intestinal mucin, supporting the notion that its high abundance in the gut may lead to defects in the intestinal barrier function (27). In the US, it was shown that compared to a control group, *A. muciniphila* was more abundant in one cluster of patients with ERA than another (29). However, in a study conducted in Europe, *A. muciniphila* was found to constitute only 1%–3% of the intestinal microbiota, a lower proportion than in the US. It has been emphasised that regional differences in the microbiota may exist and that patients in the US are more sensitive to the pro-inflammatory effects of *A. muciniphila* (62). It has been noted that *Akkermansia* spp. are pro-atherogenic bacteria. Although an increase in *Akkermansia* abundance has been reported in patients with ERA, no significant association with HLA-B27 status has been identified (42). In a study examining the faecal microbiota of children with JIA (both with and without ERA) and healthy children, *Akkermansia* spp. were not detected; instead, the researchers reported a correlation between HLA-B27 and different bacterial genera (24). The presence of *A. muciniphila*, a mucolytic bacterial species, in the intestines of HLA-B27 transgenic rats has been found to correlate with the local expression of pro-inflammatory cytokines such as interferon-*γ*, IL-17A and IL-23 and with the development of ERA, as observed in subgroups of children with ERA (63). *A. muciniphila* breaks down mucin in the gut, thereby increasing mucosal permeability, facilitating the passage of enteric bacteria into the mucosa and their access to the intestinal immune system and triggering pro-inflammatory events (27, 63–65). *A. muciniphila* is considered a commensal microorganism and does not cause inflammatory diseases; however, by breaking down intestinal mucin and increasing intestinal permeability, it disrupts the tolerogenic balance between the mucosal immune system and the intestinal microbiota, thus contributing to arthritic inflammation (27). Animal studies have shown that certain bacteria, such as *Bacteroides*, *Enterobacteriaceae*, *Porphyromonas*, *A. muciniphila* and *Clostridium ramosum*, are associated with increased mucosal inflammation in colitis (66). Some studies report that the abundance of *A. muciniphila* is reduced to almost negligible levels in patients with AS and PsA (49, 56, 67). Although an increase in *Akkermansia* has been found in the faeces of paediatric JIA patients, a reduction has been reported in adults and in those with paediatric IBD. It is unclear whether reduced *Akkermansia* abundance in IBD is a cause or a consequence. A decrease in goblet cells and mucin levels may lead to reduced *Akkermansia* abundance in individuals with IBD. Mucin serves as an energy substrate for *Akkermansia*. Interestingly, animal data suggest that *A. muciniphila* acts as a facilitator in arthritis models in mice and as a therapeutic agent in colitis models; while increasing bacterial invasiveness, it alleviates colitis (68–70). In a 2019 study, Stoll et al. reported that colonisation by *A. muciniphila* alone did not cause an increase in arthritis severity; however, its addition to a normal microbiota obtained from a healthy child not only increased arthritis severity but also reduced intestinal mucin content (70). Numerous studies have indicated that *A. muciniphila* plays a significant role in the development of inflammatory arthritis, and in our study, we found it to be present in higher abundance in the patient group compared to the control group. However, as we have not come across any studies on the relationship between JIA and gut microbiota in our country of Türkiye, it is likely that our different geographical conditions influenced this result.

The number of studies on the abundance of *Megasphaera* in stool samples from patients with arthritis is limited; however, a positive correlation between *Megasphaera* and the development of arthritis in patients with RA has been reported (71). In particular, it has been shown to be more abundant in the stool samples of treatment-resistant RA patients compared to those who respond to treatment (41). Tejesvi et al. (22), however, detected *Eubacterium biforme* and *Megasphaera* only in patients with JIA.

It has been reported that intestinal dysbiosis is associated with uveitis, for which the Bacillales order and the genus *Holdemanella* are risk factors, and that this association is mediated by blood metabolites (72). A positive correlation has been noted between an increase in *Holdemanella* in the gut microbiota and RA (73). However, another study examining the causal effect of the gut microbiota on JIA highlighted that *Holdemanella* and *Catenibacterium* reduce the risk of JIA and exert a protective effect; it also emphasised the need for further research due to findings contradicting the existing literature (74). In our study, the higher abundance of *Catenibacterium* and *Holdemanella* in the patient group compared to the control group suggests that these bacteria may be associated with JIA development. A study reported that an increase in the *Howardella* genus within the faecal microbiota may reduce the risk of developing AS (75). In our study, an increase in the *Howardella* genus was observed in the JIA group. However, further studies are needed to investigate the effect of changes in the faecal microbiota—specifically regarding the *Catenibacterium*, *Holdemanella* and *Howardella* genera—on JIA.

Some studies investigating the relationship between the abundance of the Clostridiaceae family in faecal microbial composition and inflammatory arthritis have reached different conclusions. Tejesvi et al. did not detect *Clostridium butyricum* or *Clostridium difficile* in children with JIA, observing them only in a control group (22). Stoll et al. observed a reduction in *Faecalibacterium prausnitzii* (*F.prausnitzii*) from the Clostridiaceae family in patients with JIA, while Stebbings et al. noted a decrease in *Clostridium leptum* from the same family in patients with AS compared to a control group (42, 47). Paola et al. found lower abundance of Clostridiaceae in the JIA-ERA group compared to a control group but higher abundance of Clostridium cluster XIVb, which plays a role in colitis and arthritis (24). They noted that differential abundances of Bilophila, Clostridium cluster XIVb, Oscillibacter and Parvimonas distinguished JIA patients carrying the HLA-B27 allele. Although there was a reduction in Clostridiaceae in all JIA patients [ERA and non-ERA (nERA)] compared with controls, a significant difference was observed only between JIA-ERA patients and controls. At the genus level, they also reported that Faecalibacterium, from the same family, was reduced in JIA-nERA patients compared to JIA-ERA patients and the control group, although this difference was not statistically significant. In a study examining only ERA patients, an increase was observed in Clostridium cluster XI, which includes *C. difficile*, among HLA-B27-positive patients (76). In another study, a decrease in Clostridiaceae was observed only in the ERA subgroup among JIA subgroups. At the genus level, although not statistically significant, an increase in *Clostridium* was observed in ERA patients, while a decrease in Faecalibacterium was noted in oligoarticular JIA patients (15). In patients with JIA, the following has been emphasised: the abundance of the genera *Anaerostipes*, *Dialister*, *Lachnospira* and *Roseburia* is reduced; these belong to the order *Clostridiales*; they produce SCFAs; and a reduction in their abundance correlates with more severe clinical findings (16, 77–80). By enhancing the Th17 immune response, monocolonisation of the gut by filamentous bacteria of the *Clostridium* genus leads to flareups of autoimmune conditions. In addition to microbial metabolites, certain specific bacteria such as *Clostridia*, *B. fragilis* and *F. prausnitzii* play a direct role in Treg induction (81–83). In our study, a reduction was observed in the Clostridiales order and the Clostridiaceae family, consistent with the literature.

*Lactobacillus* spp. strengthen intestinal barrier function by inducing the production of anti-inflammatory cytokines in the gut (84). It has been demonstrated that the administration of *Lactobacillus* subspecies suppresses the development of experimentally induced arthritis and alleviates disease activity in RA (85–87). Parisa Ahmadi et al. reported that the use of *Lactobacillus delbrueckii* and *Lactobacillus rhamnosus* supports anti-inflammatory effects in RA (74). However, it has been reported that Gram-positive bacteria, including *Lactobacillus* sp., are pathogenic in animal models of RA and that their abundance in the gut microbiota increases with disease activity (38). In studies of RA patients, an abundance of *Lactobacillus* has been noted in HLA-B27-positive individuals, and the arthritogenic potential of the cell wall peptidoglycan structure has been observed (76). An increase in the Lactobacillaceae family has also been demonstrated in HLA-B27-positive JIA patients (15, 24).

The main limitation of our study is the age imbalance between the patient and control groups. The control group was younger than the patient group. Considering the dynamic nature of gut microbiota development in early childhood, some of the observed differences in microbial composition could be associated with age-dependent maturation processes rather than being exclusively attributable to disease status. However, the participants showed a distribution above 2 years of age. Given that the faecal microbiota develops to a degree resembling the adult microbiota from birth to 2 years of age and stabilises in a manner similar to that of adults after 2 years of age. As in adults, Firmicutes and Bacteroidetes were found to be the two most prevalent phyla in both the patient and control groups. Given that the Firmicutes/Bacteroidetes ratio increases with age, the fact that this ratio was higher in the control group than in the patient group suggests that the faecal microbial composition had developed in a manner similar to that of adults. Despite, the findings should be validated in larger age-matched cohorts.

Other limitations include the fact that the study was conducted at a single centre with a limited number of patients and healthy children and that its findings represent only one region of our country (Türkiye). Furthermore, the cross-sectional design of the study and the absence of multivariable confounder-adjusted microbiota models, and lack of functional validation such as metagenomic, metabolomic or cytokine-based analyses should be considered when interpreting the results. These factors limit the generalisability of the findings and should be considered when interpreting the results.

Despite these limitations, our study has several strengths: the inclusion of newly diagnosed patients who had not yet received treatment, the fact that the study was conducted on a specific JIA group (namely the oligoarticular JIA group) and the assessment of relevant demographic and environmental variables, and the evaluation of a Türkiye-based cohort.

To the best of our knowledge, our study is the first of its kind conducted on this subject in Türkiye. Given that microbiota composition is influenced by geographical characteristics, our study also contributes to the literature regarding the faecal composition of JIA patients in our country. Moreover, it clarifies the need for more comprehensive, functionally validated studies in Türkiye and other geographical regions, with subsequent validation of the findings in larger, age-matched cohorts.

## 5 Conclusion

Our study supports the finding that the faecal microbial composition of children with JIA and that of healthy children differ. Diversity analyses revealed that the groups differed in terms of species richness in faecal microbial communities as well as in the presence/absence of species, which was attributed to rare species. However, their abundances were similar. Dominant bacteria were present in similar proportions, and their distributions were similar. The core microbiota was similar. However, there is a need for more comprehensive studies involving a larger number of patients and children across different geographical regions.

The present study provides insights into gut microbiota composition in children with JIA and contributes to the growing body of evidence regarding microbiota–disease associations. Nevertheless, given the substantial age difference between the groups and the inclusion of younger children in the control group, the observed microbial variations should be interpreted cautiously, as age-related microbiota maturation may have contributed to some of these differences. Future multicentre studies involving larger, age-matched cohorts from Türkiye and diverse geographical regions are needed to validate these findings and further elucidate the relationship between gut microbiota composition and disease status.

## Data Availability

The original contributions presented in the study are publicly available in the NCBI Sequence Read Archive (SRA). This data can be found under BioProject accession number PRJNA1481864.

## References

[1] Wasim R Sumaiya Ahmad A Anwar A Salman A. Microbial imbalance in the gut: a new frontier in rheumatoid arthritis research. Inflammopharmacology. (2025) 33:2277–91. doi: 10.1007/s10787-025-01737-7 40220199

[2] Jin L Xiao J Luo Y Duan L Gong Y Li Y . Exploring gut microbiota in systemic lupus erythematosus: insights and biomarker discovery potential. Clin Rev Allergy Immunol. (2025) 68:42. doi: 10.1007/s12016-025-09051-4, 40216660

[3] Fang L Ning L. Recent advances in gut microbiota and thyroid disease: pathogenesis and therapeutics in autoimmune, neoplastic, and nodular conditions. Front Cell Infect Microbiol. (2024) 14:1465928. doi: 10.3389/fcimb.2024.1465928, 39776440 PMC11703873

[4] Li J Xie Z Yang L Guo K Zhou Z. The impact of gut microbiome on immune and metabolic homeostasis in type 1 diabetes: clinical insights for prevention and treatment strategies. J Autoimmun. (2025) 151:103371. doi: 10.1016/j.jaut.2025.103371, 39883994

[5] Lionetti P Pupi A Veltroni M Fonda C Cavicchi MC Azzari C . Evidence of subclinical intestinal inflammation by 99m technetium leukocyte scintigraphy in patients with HLA-B27 positive juvenile onset active spondyloarthropathy. J Rheumatol. (2000) 27:1538–41. 10852286

[6] van Dijkhuizen EHP Del Chierico F Malattia C Russo A Pires Marafon D Ter Haar NM . Microbiome analytics of the gut microbiota in patients with juvenile idiopathic arthritis: a longitudinal observational cohort study. Arthritis Rheumatol. (2019) 71:1000–10. doi: 10.1002/art.40827, 30592383 PMC6593809

[7] Scher JU Sczesnak A Longman RS Segata N Ubeda C Bielski C . Expansion of intestinal *Prevotella copri* correlates with enhanced susceptibility to arthritis. eLife. (2013) 2:e01202. doi: 10.7554/eLife.01202, 24192039 PMC3816614

[8] Scher JU Scher JU Ubeda C Artacho A Attur M Isaac S . Decreased bacterial diversity characterizes the altered gut microbiota in patients with psoriatic arthritis, resembling dysbiosis in inflammatory bowel disease. Arthritis Rheumatol. (2015) 67:128–39. doi: 10.1002/art.38892, 25319745 PMC4280348

[9] Costello ME Ciccia F Willner D Warrington N Robinson PC Gardiner B . Brief report: intestinal dysbiosis in ankylosing spondylitis. Arthritis Rheumatol. (2015) 67:686–91. doi: 10.1002/art.38967, 25417597

[10] Brandtzaeg P. Function of mucosa-associated lymphoid tissue in antibody formation. Immunol Investig. (2010) 39:303–55. doi: 10.3109/08820131003680369, 20450282

[11] Li C Yao J Yang C Yu S Yang Z Wang L . Gut microbiota-derived short chain fatty acids act as mediators of the gut-liver-brain axis. Metab Brain Dis. (2025) 40:122. doi: 10.1007/s11011-025-01554-5, 39921774

[12] Usami M Kishimoto K Ohata A Miyoshi M Aoyama M Fueda Y . Butyrate and trichostatin a attenuate nuclear factor kappaB activation and tumor necrosis factor alpha secretion and increase prostaglandin E2 secretion in human peripheral blood mononuclear cells. Nutr Res. (2008) 28:321–8. doi: 10.1016/j.nutres.2008.02.012, 19083427

[13] Louis P Flint HJ. Diversity, metabolism and microbial ecology of butyrate-producing bacteria from the human large intestine. FEMS Microbiol Lett. (2009) 294:1–8. doi: 10.1111/j.1574-6968.2009.01514.x, 19222573

[14] Lei Y Tang L Liu S Hu S Wu L Liu Y . Parabacteroides produces acetate to alleviate heparanase-exacerbated acute pancreatitis through reducing neutrophil infiltration. Microbiome. (2021) 9:115. doi: 10.1186/s40168-021-01065-2, 34016163 PMC8138927

[15] De Filippo C Di Paola M Giani T Tirelli F Cimaz R. Gut microbiota in children and altered profiles in juvenile idiopathic arthritis. J Autoimmun. (2019) 98:1–12. doi: 10.1016/j.jaut.2019.01.001, 30638708

[16] Qian X Liu YX Ye X Zheng W Lv S Mo M . Gut microbiota in children with juvenile idiopathic arthritis: characteristics, biomarker identification, and usefulness in clinical prediction. BMC Genomics. (2020) 21:286. doi: 10.1186/s12864-020-6703-0, 32264859 PMC7137182

[17] Hui W Yu D Cao Z Zhao X. Butyrate inhibit collagen-induced arthritis via Treg/IL-10/Th17 axis. Int Immunopharmacol. (2019) 68:226–33. doi: 10.1016/j.intimp.2019.01.018, 30660077

[18] Wang H Cai Y Wu W Zhang M Dai Y Wang Q. Exploring the role of gut microbiome in autoimmune diseases: a comprehensive review. Autoimmun Rev. (2024) 23:103654. doi: 10.1016/j.autrev.2024.103654, 39384149

[19] Berntson L Öman A Engstrand L Dicksved J. A pilot study investigating faecal microbiota after two dietary interventions in children with juvenile idiopathic arthritis. Curr Microbiol. (2022) 79:215. doi: 10.1007/s00284-022-02899-1, 35672613 PMC9174309

[20] Rigante D Bosco A Esposito S. The Etiology of juvenile idiopathic arthritis. Clin Rev Allergy Immunol. (2015) 49:253–61. doi: 10.1007/s12016-014-8460-9, 25384710

[21] Verwoerd A Ter Haar NM de Roock S Vastert SJ Bogaert D. The human microbiome and juvenile idiopathic arthritis. Pediatr Rheumatol Online J. (2016) 14:55. doi: 10.1186/s12969-016-0114-4, 27650128 PMC5028952

[22] Tejesvi MV Arvonen M Kangas SM Keskitalo PL Pirttilä AM Karttunen TJ . Faecal microbiome in new-onset juvenile idiopathic arthritis. Eur J Clin Microbiol Infect Dis. (2016) 35:363–70. doi: 10.1007/s10096-015-2548-x, 26718942

[23] Arvonen M Berntson L Pokka T Karttunen TJ Vähäsalo P Stoll ML. Gut microbiota-host interactions and juvenile idiopathic arthritis. Pediatr Rheumatol Online J. (2016) 4:44. doi: 10.1186/s12969-016-0104-6 PMC4957868 27448997

[24] Di Paola M Cavalieri D Albanese D Sordo M Pindo M Donati C . Alteration of fecal microbiota profiles in juvenile idiopathic arthritis. Associations with HLA-B27 allele and disease status. Front Microbiol. (2016) 7:1703. doi: 10.3389/fmicb.2016.01703, 27833598 PMC5080347

[25] Berntson L Agback P Dicksved J. Changes in fecal microbiota and metabolomics in a child with juvenile idiopathic arthritis (JIA) responding to two treatment periods with exclusive enteral nutrition (EEN). Clin Rheumatol. (2016) 35:1501–6. doi: 10.1007/s10067-016-3238-5, 27021336

[26] Arvonen M Vähäsalo P Turunen S Salo HM Mäki M Laurila K . Altered expression of intestinal human leucocyte antigen D-related and immune signalling molecules in juvenile idiopathic arthritis. Clin Exp Immunol. (2012) 170:266–73. doi: 10.1111/j.1365-2249.2012.04663.x, 23121667 PMC3518886

[27] Derrien M Vaughan EE Plugge CM de Vos WM. *Akkermansia muciniphila* gen. Nov., sp. nov., a human intestinal mucin-degrading bacterium. Int J Syst Evol Microbiol. (2004) 54:1469–76. doi: 10.1099/ijs.0.02873-0, 15388697

[28] Öman A Dicksved J Engstrand L Berntson L. Fecal microbiota in untreated children with juvenile idiopathic arthritis: a comparison with healthy children and healthy siblings. J Rheumatol. (2021) 48:1589–95. doi: 10.3899/jrheum.200551, 33262301

[29] Mielants H Veys EM Cuvelier C De Vos M Goemaere S Maertens M . Gut inflammation in children with late onset pauciarticular juvenile chronic arthritis and evolution to adult spondyloarthropathy–a prospective study. J Rheumatol. (1993) 20:1567–72. 8164217

[30] Round JL Mazmanian SK. The gut microbiota shapes intestinal immune responses during health and disease. Nat Rev Immunol. (2009) 9:313–23. doi: 10.1038/nri2515, 19343057 PMC4095778

[31] Tailford LE Crost EH Kavanaugh D Juge N. Mucin glycan foraging in the human gut microbiome. Front Genet. (2015) 6:81. doi: 10.3389/fgene.2015.00081, 25852737 PMC4365749

[32] Chang PV Hao L Offermanns S Medzhitov R. The microbial metabolite butyrate regulates intestinal macrophage function via histone deacetylase inhibition. Proc Natl Acad Sci USA. (2014) 111:2247–52. doi: 10.1073/pnas.1322269111, 24390544 PMC3926023

[33] Robertson RC Manges AR Finlay BB Prendergast AJ. The human microbiome and child growth - first 1000 days and beyond. Trends Microbiol. (2019) 27:131–47. doi: 10.1016/j.tim.2018.09.008, 30529020

[34] Rinninella E Raoul P Cintoni M Franceschi F Miggiano GAD Gasbarrini A . What is the healthy gut microbiota composition? A changing ecosystem across age, environment, diet, and diseases. Microorganisms. (2019) 7:14. doi: 10.3390/microorganisms7010014, 30634578 PMC6351938

[35] Budzinski L Sempert T Lietz L Maier R Kang GU von Stuckrad ASL . Age-stratification reveals age-specific intestinal microbiota signatures in juvenile idiopathic arthritis. Mol Cell Pediatr. (2024) 11:12. doi: 10.1186/s40348-024-00186-6, 39653980 PMC11628465

[36] Dong YQ Wang W Li J Ma MS Zhong LQ Wei QJ . Characterization of microbiota in systemic-onset juvenile idiopathic arthritis with different disease severities. World J Clin Cases. (2019) 7:2734–45. doi: 10.12998/wjcc.v7.i18.2734, 31616689 PMC6789395

[37] Ricciuto A Sherman PM Laxer RM. Gut microbiota in chronic inflammatory disorders: a focus on pediatric inflammatory bowel diseases and juvenile idiopathic arthritis. Clin Immunol. (2020) 215:108415. doi: 10.1016/j.clim.2020.108415, 32278875

[38] Zhang X Zhang D Jia H Feng Q Wang D Liang D . The oral and gut microbiomes are perturbed in rheumatoid arthritis and partly normalized after treatment. Nat Med. (2015) 21:895–905. doi: 10.1038/nm.3914, 26214836

[39] Breban M Tap J Leboime A Said-Nahal R Langella P Chiocchia G . Faecal microbiota study reveals specific dysbiosis in spondyloarthritis. Ann Rheum Dis. (2017) 76:1614–22. doi: 10.1136/annrheumdis-2016-211064, 28606969

[40] Öman A Dicksved J Engstrand L Berntson L. Fecal microbiota in children with juvenile idiopathic arthritis treated with methotrexate or etanercept. Pediatr Rheumatol Online J. (2021) 19:55. doi: 10.1186/s12969-021-00542-0, 33902613 PMC8077782

[41] Ruiz-Limón P Mena-Vázquez N Moreno-Indias I Lisbona-Montañez JM Mucientes A Manrique-Arija S . Gut dysbiosis is associated with difficult-to-treat rheumatoid arthritis. Front Med (Lausanne). (2025) 11:1497756. doi: 10.3389/fmed.2024.1497756, 39886456 PMC11781114

[42] Stoll ML Kumar R Morrow CD Lefkowitz EJ Cui X Genin A . Altered microbiota associated with abnormal humoral immune responses to commensal organisms in enthesitis-related arthritis. Arthritis Res Ther. (2014) 16:486. doi: 10.1186/s13075-014-0486-0, 25434931 PMC4272554

[43] Stoll ML Weiss PF Weiss JE Nigrovic PA Edelheit BS Bridges SL Jr . Age and fecal microbial strain-specific differences in patients with spondyloarthritis. Arthritis Res Ther. (2018) 20:14. doi: 10.1186/s13075-018-1510-6, 29382366 PMC5791354

[44] Muller PH De Meij TG Westedt M de Groot EF Allaart CF Brinkman DM . Disturbance of microbial core species in new-onset juvenile idiopathic arthritis. J Pediatr Infect Dis J. (2017) 12:131–5. doi: 10.1055/s-0037-1601340

[45] Stoll ML DeQuattro K Li Z Sawhney H Weiss PF Nigrovic PA . Impact of HLA-B27 and disease status on the gut microbiome of the offspring of ankylosing spondylitis patients. Children (Basel). (2022) 9:569. doi: 10.3390/children9040569 35455612 PMC9030797

[46] Maeda Y Kurakawa T Umemoto E Motooka D Ito Y Gotoh K . Dysbiosis contributes to arthritis development via activation of autoreactive T cells in the intestine. Arthritis Rheumatol. (2016) 68:2646–61. doi: 10.1002/art.39783, 27333153

[47] Stebbings S Munro K Simon MA Tannock G Highton J Harmsen H . Comparison of the faecal microflora of patients with ankylosing spondylitis and controls using molecular methods of analysis. Rheumatology (Oxford). (2002) 41:1395–401. doi: 10.1093/rheumatology/41.12.1395, 12468819

[48] Tito RY Cypers H Joossens M Varkas G Van Praet L Glorieus E . Brief report: *Dialister* as a microbial marker of disease activity in spondyloarthritis. Arthritis Rheumatol. (2017) 69:114–21. doi: 10.1002/art.39802 27390077

[49] Klingberg E Magnusson MK Strid H Deminger A Ståhl A Sundin J . A distinct gut microbiota composition in patients with ankylosing spondylitis is associated with increased levels of fecal calprotectin. Arthritis Res Ther. (2019) 21:248. doi: 10.1186/s13075-019-2018-4, 31771630 PMC6880506

[50] Wen C Zheng Z Shao T Liu L Xie Z Le Chatelier E . Quantitative metagenomics reveals unique gut microbiome biomarkers in ankylosing spondylitis. Genome Biol. (2017) 18:142. doi: 10.1186/s13059-017-1271-6, 28750650 PMC5530561

[51] Chen Z Qi J Wei Q Zheng X Wu X Li X . Variations in gut microbial profiles in ankylosing spondylitis: disease phenotype-related dysbiosis. Ann Transl Med. (2019) 7:571. doi: 10.21037/atm.2019.09.41, 31807552 PMC6861740

[52] Lin A Bik EM Costello EK Dethlefsen L Haque R Relman DA . Distinct distal gut microbiome diversity and composition in healthy children from Bangladesh and the United States. PLoS One. (2013) 8:e53838. doi: 10.1371/journal.pone.0053838 23349750 PMC3551965

[53] Aggarwal A Sarangi AN Gaur P Shukla A Aggarwal R. Gut microbiome in children with enthesitis-related arthritis in a developing country and the effect of probiotic administration. Clin Exp Immunol. (2017) 187:480–9. doi: 10.1111/cei.12900, 27861762 PMC5290238

[54] Morotomi M Nagai F Sakon H Tanaka R. *Dialister succinatiphilus* sp. nov. and *Barnesiella intestinihominis* sp. nov., isolated from human faeces. Int J Syst Evol Microbiol. (2008) 58:2716–20. doi: 10.1099/ijs.0.2008/000810-0, 19060046

[55] Kindgren E Ahrens AP Triplett EW Ludvigsson J. Infant gut microbiota and environment associate with juvenile idiopathic arthritis many years prior to disease onset, especially in genetically vulnerable children. EBioMedicine. (2023) 93:104654. doi: 10.1016/j.ebiom.2023.104654, 37329576 PMC10279551

[56] Bixio R Bertelle D Bertoldo E Morciano A Rossini M. The potential pathogenic role of gut microbiota in rheumatic diseases: a human-centred narrative review. Intern Emerg Med. (2024) 19:891–900. doi: 10.1007/s11739-023-03496-1, 38141117

[57] Rau M Rehman A Dittrich M Groen AK Hermanns HM Seyfried F . Fecal SCFAs and SCFA-producing bacteria in gut microbiome of human NAFLD as a putative link to systemic T-cell activation and advanced disease. United European Gastroenterol J. (2018) 6:1496–507. doi: 10.1177/2050640618804444, 30574320 PMC6297934

[58] Moschen AR Gerner RR Wang J Klepsch V Adolph TE Reider SJ . Lipocalin 2 protects from inflammation and tumorigenesis associated with gut microbiota alterations. Cell Host Microbe. (2016) 19:455–69. doi: 10.1016/j.chom.2016.03.007, 27078067

[59] Zuo K Li J Li K Hu C Gao Y Chen M . Disordered gut microbiota and alterations in metabolic patterns are associated with atrial fibrillation. Gigascience. (2019) 8:giz058. doi: 10.1093/gigascience/giz058, 31149718 PMC6543127

[60] Bangsgaard Bendtsen KM Krych L Sørensen DB Pang W Nielsen DS Josefsen K . Gut microbiota composition is correlated to grid floor induced stress and behavior in the BALB/c mouse. PLoS One. (2012) 7:e46231. doi: 10.1371/journal.pone.0046231, 23056268 PMC3462757

[61] Kaur H Das C Mande SS. In silico analysis of putrefaction pathways in Bacteria and its implication in colorectal Cancer. Front Microbiol. (2017) 8:2166. doi: 10.3389/fmicb.2017.02166, 29163445 PMC5682003

[62] Derrien M Collado MC Ben-Amor K Salminen S de Vos WM. The mucin degrader *Akkermansia muciniphila* is an abundant resident of the human intestinal tract. Appl Environ Microbiol. (2008) 74:1646–8. doi: 10.1128/AEM.01226-07, 18083887 PMC2258631

[63] Asquith MJ Stauffer P Davin S Mitchell C Lin P Rosenbaum JT. Perturbed mucosal immunity and dysbiosis accompany clinical disease in a rat model of Spondyloarthritis. Arthritis Rheumatol. (2016) 68:2151–62. doi: 10.1002/art.39681, 26992013 PMC5542398

[64] Wells CL van de Westerlo EM Jechorek RP Feltis BA Wilkins TD Erlandsen SL. *Bacteroides fragilis* enterotoxin modulates epithelial permeability and bacterial internalization by HT-29 enterocytes. Gastroenterology. (1996) 110:1429–37. doi: 10.1053/gast.1996.v110.pm8613048, 8613048

[65] Martínez-González O Cantero-Hinojosa J Paule-Sastre P Gómez-Magán JC Salvatierra-Ríos D. Intestinal permeability in patients with ankylosing spondylitis and their healthy relatives. Br J Rheumatol. (1994) 33:644–7. doi: 10.1093/rheumatology/33.7.644, 8019793

[66] Gkouskou KK Deligianni C Tsatsanis C Eliopoulos AG. The gut microbiota in mouse models of inflammatory bowel disease. Front Cell Infect Microbiol. (2014) 4:28. doi: 10.3389/fcimb.2014.00028, 24616886 PMC3937555

[67] Zhou C Zhao H Xiao XY Chen BD Guo RJ Wang Q . Metagenomic profiling of the pro-inflammatory gut microbiota in ankylosing spondylitis. J Autoimmun. (2020) 107:102360. doi: 10.1016/j.jaut.2019.102360, 31806420

[68] Ganesh BP Klopfleisch R Loh G Blaut M. Commensal *Akkermansia muciniphila* exacerbates gut inflammation in *Salmonella Typhimurium*-infected gnotobiotic mice. PLoS One. (2013) 8:e74963. doi: 10.1371/journal.pone.0074963, 24040367 PMC3769299

[69] Bian X Wu W Yang L Lv L Wang Q Li Y . Administration of *Akkermansia muciniphila* ameliorates dextran Sulfate sodium-induced ulcerative colitis in mice. Front Microbiol. (2019) 10:2259. doi: 10.3389/fmicb.2019.02259, 31632373 PMC6779789

[70] Stoll ML Pierce MK Watkins JA Zhang M Weiss PF Weiss JE . *Akkermansia muciniphila* is permissive to arthritis in the K/BxN mouse model of arthritis. Genes Immun. (2019) 20:158–66. doi: 10.1038/s41435-018-0024-1, 29599513 PMC6153082

[71] El Menofy NG Ramadan M Abdelbary ER Ibrahim HG Azzam AI Ghit MM . Bacterial compositional shifts of gut microbiomes in patients with rheumatoid arthritis in association with disease activity. Microorganisms. (2022) 10:1820. doi: 10.3390/microorganisms10091820, 36144422 PMC9505928

[72] Xie X Ren W Zhou W Zhang X Deng X Wang X . Genetic prediction of the effect of gut microbiota on uveitis via blood metabolites: a mediated mendelian randomization investigation. Medicine (Baltimore). (2024) 103:e40922. doi: 10.1097/MD.0000000000040922, 39686482 PMC11651470

[73] Gou Y Zhang J Li C Liu Y Hui J Zhou R . Causal relationship between gut microbiota and rheumatoid arthritis: a two-sample mendelian randomisation study. Clin Exp Rheumatol. (2024) 42:166–73. doi: 10.55563/clinexprheumatol/p9ig7c, 37812479

[74] Zhang L Yang Z Zhang L Wei Y Wan L. Causal effect of gut microbiota on juvenile idiopathic arthritis: a two-sample mendelian a randomization study. J Cell Mol Med. (2024) 28:e70183. doi: 10.1111/jcmm.70183, 39473264 PMC11522359

[75] Du X Li H Zhao H Cui S Sun X Tan X. Causal relationship between gut microbiota and ankylosing spondylitis and potential mediating role of inflammatory cytokines: a mendelian randomization study. PLoS One. (2024) 19:e0306792. doi: 10.1371/journal.pone.0306792, 39083521 PMC11290680

[76] Morio F Jean-Pierre H Dubreuil L Jumas-Bilak E Calvet L Mercier G . Antimicrobial susceptibilities and clinical sources of Dialister species. Antimicrob Agents Chemother. (2007) 51:4498–501. doi: 10.1128/AAC.00538-07, 17923492 PMC2167981

[77] Bui TPN de Vos WM Plugge CM. *Anaerostipes rhamnosivorans* sp. nov., a human intestinal, butyrate-forming bacterium. Int J Syst Evol Microbiol. (2014) 64:787–93. doi: 10.1099/ijs.0.055061-0, 24215821

[78] Schwiertz A Hold GL Duncan SH Gruhl B Collins MD Lawson PA . *Anaerostipes caccae* gen. Nov., sp. nov., a new saccharolytic, acetate-utilising, butyrate-producing bacterium from human faeces. Syst Appl Microbiol. (2002) 25:46–51. doi: 10.1078/0723-2020-00096, 12086188

[79] Meehan CJ Beiko RG. A phylogenomic view of ecological specialization in the Lachnospiraceae, a family of digestive tract-associated bacteria. Genome Biol Evol. (2014) 6:703–13. doi: 10.1093/gbe/evu050, 24625961 PMC3971600

[80] Tamanai-Shacoori Z Smida I Bousarghin L Loreal O Meuric V Fong SB . Roseburia spp.: a marker of health? Future Microbiol. (2017) 12:157–70. doi: 10.2217/fmb-2016-0130, 28139139

[81] Wu HJ Ivanov II Darce J Hattori K Shima T Umesaki Y . Gut-residing segmented filamentous bacteria drive autoimmune arthritis via T helper 17 cells. Immunity. (2010) 32:815–27. doi: 10.1016/j.immuni.2010.06.001, 20620945 PMC2904693

[82] Round JL Mazmanian SK. Inducible Foxp3+ regulatory T-cell development by a commensal bacterium of the intestinal microbiota. Proc Natl Acad Sci USA. (2010) 107:12204–9. doi: 10.1073/pnas.0909122107, 20566854 PMC2901479

[83] Smith PM Howitt MR Panikov N Michaud M Gallini CA Bohlooly YM . The microbial metabolites, short-chain fatty acids, regulate colonic Treg cell homeostasis. Science. (2013) 341:569–73. doi: 10.1126/science.1241165 23828891 PMC3807819

[84] Martín R Chain F Miquel S Natividad JM Sokol H Verdu EF . Effects in the use of a genetically engineered strain of *Lactococcus lactis* delivering in situ IL-10 as a therapy to treat low-grade colon inflammation. Hum Vaccin Immunother. (2014) 10:1611–21. doi: 10.4161/hv.28549, 24732667 PMC5396252

[85] Kano H Kaneko T Kaminogawa S. Oral intake of *Lactobacillus delbrueckii* subsp. bulgaricus OLL1073R-1 prevents collagen-induced arthritis in mice. J Food Prot. (2002) 65:153–60. doi: 10.4315/0362-028X-65.1.153, 11808787

[86] So JS Kwon HK Lee CG Yi HJ Park JA Lim SY . *Lactobacillus casei* suppresses experimental arthritis by down-regulating T helper 1 effector functions. Mol Immunol. (2008) 45:2690–9. doi: 10.1016/j.molimm.2007.12.010, 18243320

[87] Vaghef-Mehrabany E Alipour B Homayouni-Rad A Sharif SK Asghari-Jafarabadi M Zavvari S. Probiotic supplementation improves inflammatory status in patients with rheumatoid arthritis. Nutrition. (2014) 30:430–5. doi: 10.1016/j.nut.2013.09.007, 24355439

